# A rare presentation of gastric ischemia manifesting as gastric pneumatosis in the context of amphetamine use

**DOI:** 10.1093/jscr/rjag535

**Published:** 2026-06-29

**Authors:** Muhammed Yaman Swied, Muhammad Anas Abdulrazzak, Obada Daaboul, Muaataz Azzawi, Michael Maitar

**Affiliations:** Department of Internal Medicine, Southern Illinois University School of Medicine, 801 N. Rutledge St., Springfield, IL 62702, United States; Department of Internal Medicine, Southern Illinois University School of Medicine, 801 N. Rutledge St., Springfield, IL 62702, United States; Department of Internal Medicine, Southern Illinois University School of Medicine, 801 N. Rutledge St., Springfield, IL 62702, United States; Department of Gastroenterology, Southern Illinois University School of Medicine, 801 N. Rutledge St., Springfield, IL 62702, United States; Department of Gastroenterology, Southern Illinois University School of Medicine, 801 N. Rutledge St., Springfield, IL 62702, United States

**Keywords:** gastric ischemia, gastric pneumatosis, amphetamine

## Abstract

Gastric pneumatosis is a rare condition characterized by gas within the stomach wall and can be associated with gastric ischemia. Gastric ischemia is uncommon due to the stomach’s rich vascular supply but carries an increased risk of mortality. We present a rare case of a patient with amphetamine use disorder who was found to have gastric pneumatosis in the setting of gastric ischemia.

## Introduction

Gastric ischemia is a rare condition caused by diffuse or localized vascular insufficiency. Predisposing factors include, but are not limited to, systemic hypoperfusion (e.g. shock), vasculitis, or disseminated intravascular coagulation; other mechanisms, such as arterial occlusion or drug-induced vasospasm, have also been described. True gastric ischemia is infrequently reported and often under-recognized clinically due to the stomach’s rich blood supply. Patients with gastric ischemia typically present with acute and severe abdominal pain, nausea, vomiting, gastrointestinal bleeding, or signs of sepsis. Endoscopy may show mucosal erythema, ulceration, or necrosis, while imaging can reveal gastric wall thickening, pneumatosis, or portal venous gas. Rapid diagnosis and management are essential due to the condition’s high mortality rate. We are reporting a rare case of gastric ischemia manifesting as gastric pneumatosis in the setting of amphetamine use.

## Case report

**Figure 1:**
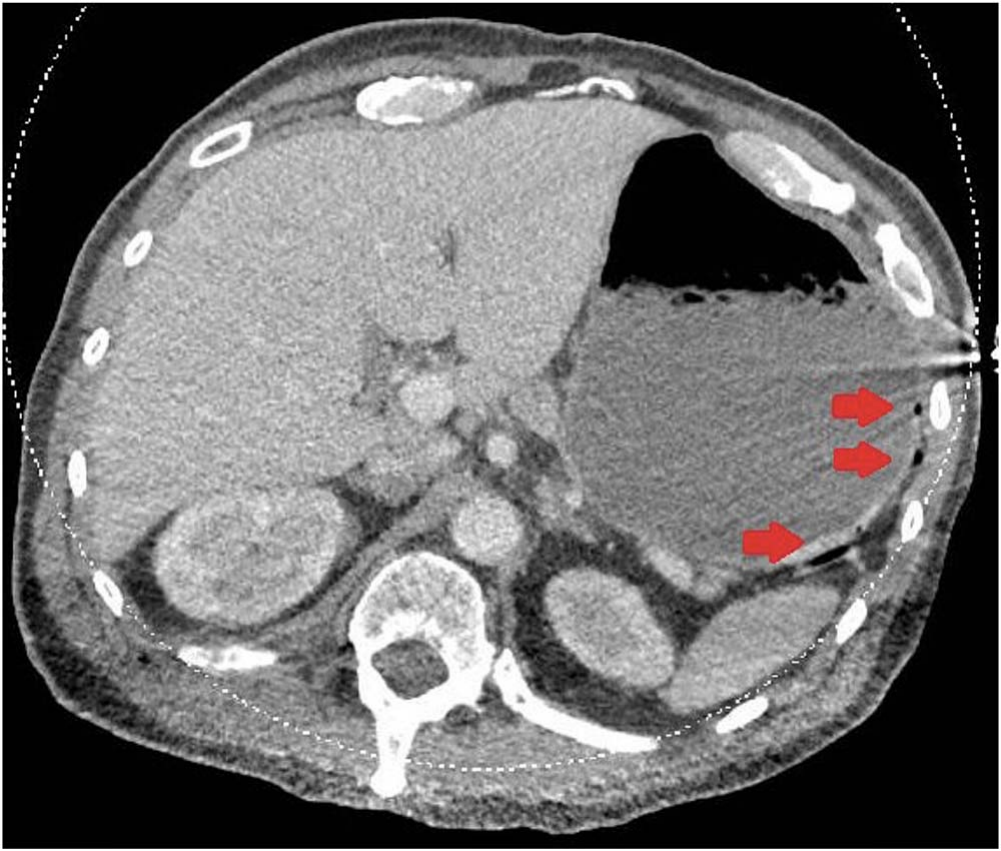
Axial CT image of the abdomen demonstrating gastric pneumatosis, indicated by red arrows.

A 68-year-old man with amphetamine use disorder presented with abdominal pain, diarrhea, and hematemesis. The physical exam was unremarkable except for diffuse abdominal tenderness. The patient was found to have hypotension requiring fluid resuscitation and vasopressors. Lactic acid was 1.2 mmol/l. Urine drug screen was positive for amphetamine. Abdominal computed tomography (CT) showed a small amount of pneumatosis of the posterior wall of the stomach and adjacent mesenteric venous gas, concerning for ischemia and infarction (Fig. 1). Triple-phase abdominal CT showed chronic 50% luminal stenosis of the celiac and superior mesenteric artery origins. A nasogastric tube was placed for decompression, and the patient was started on intravenous PPI, ceftriaxone, and metronidazole. Due to refractory shock and hemodynamic instability, the patient underwent exploratory laparotomy and was found to have palpable gastric pneumatosis with no gross evidence of gastric ischemia, as it was likely mucosal or submucosal rather than transmural insult, but intraoperative esophagogastroscopy showed multiple patchy ischemic changes of the posterior stomach wall and gastroesophageal junction. The patient was managed conservatively, achieved hemodynamic stability postoperatively, and reported symptomatic improvement.

## Discussion

Gastric pneumatosis, characterized by air within the stomach wall, is a rare condition associated with adverse clinical outcomes. A 2020 review identified 111 publications from the preceding 25 years, most of which were single-case reports [1]. Gastric ischemia is the most common mechanism underlying gastric pneumatosis [1]. Other mechanisms include gastric intramural infection, also known as emphysematous gastritis, mucosal disruption, and the extension of supradiaphragmatic air [1].

Gastric ischemia is characterized by insufficient blood flow to the stomach, resulting in tissue hypoxia and mucosal injury. This condition is rare due to the stomach’s extensive collateral blood supply [2, 3]. Gastric ischemia may result from systemic hypoperfusion (such as shock or severe hypotension), local vascular occlusion (such as celiac artery occlusion or superior mesenteric artery occlusion), or mechanical obstruction (such as gastric volvulus or external compression) [2, 3]. In our case, the pathophysiology of gastric ischemia was likely multifactorial. Imaging demonstrated chronic luminal stenosis at the origins of the celiac and superior mesenteric arteries, representing a significant predisposing anatomical cause of gastric ischemia. Additionally, hemodynamic instability requiring vasopressors likely resulted in systemic hypoperfusion in the setting of underlying vascular occlusion.

Amphetamine is a potent central nervous system stimulant that increases the availability of dopamine, norepinephrine, and serotonin. Although it is prescribed for attention-deficit/hyperactivity disorder, amphetamine is also widely misused and has a high potential for addiction. Amphetamine-induced bowel ischemia has been reported in the literature, but amphetamine-induced gastric ischemia is rarely reported. Amphetamine-induced gastric ischemia is likely caused by vasoconstriction in the splanchnic circulation and disruption of microvascular flow due to the drug’s sympathomimetic properties [4, 5]. The underlying mechanism involves a surge in catecholamines that triggers alpha-1 adrenergic receptor-mediated vasospasm, leading to decreased blood flow to the gastric tissues and resulting in mucosal damage or necrosis [4, 5]. While amphetamine use could have contributed to gastric ischemia in our case through its sympathomimetic properties, it should be considered as one factor among the systemic vascular occlusion and hemodynamic instability requiring vasopressors.

Patients with gastric ischemia may present with nonspecific symptoms such as abdominal pain, nausea, vomiting, gastrointestinal bleeding, or signs of sepsis. Imaging can reveal gastric pneumatosis or portal venous air, and endoscopic findings often include mucosal congestion, erythema, ulceration, and, in severe cases, necrosis. The diagnosis of gastric ischemia is established through clinical history, imaging studies, and endoscopic evaluation. In our case, it was suspected that chronic stenosis of the celiac and superior mesenteric artery predisposed the patient to have gastric ischemia, which could be triggered by amphetamine use.

Management of gastric ischemia is primarily supportive and includes volume resuscitation, nasogastric decompression, intravenous proton pump inhibitors, and broad-spectrum antibiotics [3, 6]. Surgical intervention is reserved for complications such as perforation, necrosis, or refractory shock [7]. In this case, the patient initially received conservative management but subsequently required an exploratory laparotomy due to refractory shock.

In conclusion, we report a case of gastric ischemia presenting as gastric pneumatosis in the context of amphetamine use. This case highlights the multifactorial pathophysiology of gastric ischemia and the importance of considering this diagnosis in patients with amphetamine use disorder who present with nonspecific gastrointestinal symptoms and imaging findings suggestive of gastric pneumatosis.
